# Culture density contributes to hepatic functions of fresh human hepatocytes isolated from chimeric mice with humanized livers: Novel, long-term, functional two-dimensional *in vitro* tool for developing new drugs

**DOI:** 10.1371/journal.pone.0237809

**Published:** 2020-09-11

**Authors:** Chihiro Yamasaki, Yuji Ishida, Ami Yanagi, Yasumi Yoshizane, Yuha Kojima, Yuko Ogawa, Yutaka Kageyama, Yumiko Iwasaki, Seiichi Ishida, Kazuaki Chayama, Chise Tateno

**Affiliations:** 1 PhoenixBio Co., Ltd., Higashi-Hiroshima, Hiroshima, Japan; 2 Research Center for Hepatology and Gastroenterology, Hiroshima University, Hiroshima, Japan; 3 Department of Pharmacology, National Institute of Health Sciences, Kanagawa, Japan; 4 Department of Gastroenterology and Metabolism, Applied Life Sciences, Institute of Biomedical and Health Sciences, Hiroshima University, Higashihiroshima, Hiroshima, Japan; Universita degli Studi Di Cagliari, ITALY

## Abstract

Chimeric mice with humanized livers are considered a useful animal model for predicting human (h-) drug metabolism and toxicity. In this study, the characteristics of fresh h-hepatocytes (cFHHs, PXB-cells^®^) isolated from chimeric mice (PXB-mice^®^) were evaluated *in vitro* to confirm their utility for drug development. cFHHs cultured at high density (2.13 × 10^5^ cells/cm^2^) displayed stable production of h-albumin and cytochrome P450 (CYP) 3A activities for at least 21 days. The mRNA expression levels of 10 of 13 CYP, UDP-glucuronosyltransferase (UGT), and transporters were maintained at >10% of the levels of freshly isolated cFHHs after 21 days. From 1 week, many bile canaliculi were observed between cFHHs, and the accumulation of the multidrug resistance-associated protein and bile salt export pump substrates in these bile canaliculi was clearly inhibited by cyclosporin A. Microarray analysis of cFHHs cultured at high density and at low density (0.53 × 10^5^ cells/cm^2^) revealed that high density culture maintained high expressions of some transcription factors (HNF4α, PXR, and FXR) perhaps involved in the high CYP, UGT and transporter gene expressions of cFHHs. These results strongly suggest that cFHHs could be a novel *in vitro* tool for drug development studies.

## Introduction

Drug metabolism, transporter, and hepatotoxicity tests for new chemical entities have been performed using cryopreserved human (ch-) hepatocytes as the gold standard. Although fresh human (h-) hepatocytes retain higher metabolic activities to some extent than ch-hepatocytes [1, 2], an on-demand supply and reproducible experiments using fresh h-hepatocytes from the same donor are impossible.

Generally, when rat hepatocytes are inoculated at high density on dishes (>2 × 10^5^ cells/cm^2^), attachment efficiency decreases due to overlaying [3]. In our experience, when rat or mouse (m-) hepatocytes were inoculated at high density on collagen-coated dishes (>2×10^5^ cells/cm^2^), hepatocytes detached from the dishes because cell-cell adhesion was stronger than cell dish adhesion. Therefore, in general, fresh hepatocytes have been preconfluently inoculated for metabolism or toxicology studies. However, cytochrome P450 enzyme (CYP) mRNA expression and activity in preconfluently cultured hepatocytes promptly decline [4, 5]. Therefore, studies of drug metabolism have been performed using h-hepatocytes suspended for 1 or 2 hours. In addition, because fresh m-hepatocytes and thawed ch-hepatocytes do not remain viable for longer than 1 week in traditional two-dimensional (2D) cultures, novel 2D culture system [6], sandwich 2D cultures [7], or three-dimensional cultures are needed for long-term experiments.

In the last decade, chimeric mice with humanized livers have been developed using several host mice, including urokinase-type plasminogen activator/severe combined immunodeficiency (uPA/SCID) mice [8, 9], Fah(-/-)Rag2(-/-)Il2rg (-/-) mice [10], and NOG-Tg (Alb-UL23) 7-2/ShiJic (TK-NOG) mice [11]. We succeeded in the stable and mass production of chimeric mice with humanized livers using uPA/SCID mice (PXB-mice^®^) [8]. The mRNA expression levels of approximately 82% of h-hepatocyte genes from chimeric mice were similar to or within 2-fold of the levels in human liver [12]. Protein expression levels of phase I CYPs and transporters in chimeric mouse livers were similar to or within 4-fold of the levels in human liver [13]. In addition, we recently developed a new type of host mouse, the cDNA-uPA/SCID mouse [14]. Chimeric mice generated from cDNA-uPA/SCID mice exhibited improved characteristics over those of chimeric mice generated from uPA/SCID mice. The improvements included increased body weight and higher stable repopulation rates of h-hepatocytes [14]. Our chimeric mice have been utilized for *in vivo* drug metabolism and pharmacokinetics (DMPK) [15–17], drug-drug interaction [5, 18], and toxicology studies [19–21]. The chimeric mice also have been used for efficacy studies of chemical entities against hepatitis B virus and hepatitis C virus, because the chimeric mice are susceptible to these hepatitis viruses [22–24].

We obtained fresh h-hepatocytes (cFHHs, PXB-cells^®^) from our chimeric mice obtained by a collagenase perfusion method [1] and reported that CYP activities of suspended fresh h-hepatocytes and ch-hepatocytes from cFHHs were similar [1]. We also reported *in vitro* and *in vivo* CYP mRNA induction studies using chimeric mice and cFHHs with the same donor hepatocytes [5].

Recently, we demonstrated that cultured cFHHs were susceptible to HBV infection and that HBV-infected cFHHs can be maintained for more than 3 weeks when the cFHHs were cultured on collagen-coated dishes at a high density (2.13 × 10^5^ cells/cm^2^) [25]. Therefore, cFHHs have been used for *in vitro* efficacy studies of anti-HBV agents [25]. In those studies, we found that cFHHs can attach to collagen-coated dishes in high density cultures without the detachment seen in rodent hepatocytes and can be maintained for more than 3 weeks [25].

In the present study, high density 2D-cultures of cFHHs enabled long-term culture of h-hepatocytes maintaining CYP, UDP-glucuronosyltransferase (UGT), transporter mRNA expression and the activities of CYP3A and multidrug resistance-associated protein (MRP2) transporter and bile salt export pump (BSEP). We also evaluated differences in donors and individual chimeric mice using ch-hepatocytes isolated from three different donors. Finally, the role of the high density culture condition was determined by microarray analysis of cFHHs cultured at high and low density.

## Materials and methods

### Chemicals

Krebs–Henseleit buffer was purchased from Sigma-Aldrich (St. Louis, MO). Midazolam, and cyclosporin A (CsA) were obtained from Wako Pure Chemical Industries (Osaka, Japan). 5(6)-Carboxy-2',7'-dichlorofluorescein diacetate (CDFDA) was purchased from Thermo Fisher Scientific (Waltham, MA). N-(24-[7-(4-N,N-dimethylaminosulfonyl-2,1,3-benzoxadiazole)]amino-3α,7α,12α-trihydroxy-27-nor-5 β-cholestan-26-oyl)-2'-aminoethanesulfonate (Tauro-nor-THCA-24-DBD, DBD) was purchased from GenoMembrane (Kanagawa, Japan). All other chemicals and solvents were of the highest grade commercially available.

### Generation of chimeric mice (PXB-mice^®^)

ch-Hepatocytes, BD195 (donor A, platable, inducible, and transporter activity qualified, 2-year-old Hispanic girl), BD342 (donor B, platable, 2-year-old white girl), and BD85 (donor C, non-platable, 5-year-old African American girl) were purchased from BD Biosciences (Woburn, MA). cH-hepatocytes, IVT-JFC (donor D, platable, 1-year-old white boy) was purchased from BioIVT (Westbury, NY). These samples were obtained after written informed consent and this study was approved by Utilization of Human Tissue Ethical Committee of PhoenixBio Co., Ltd. (0028). ch-hepatocytes were thawed and transplanted into 2 to 4-week-old cDNA-uPA/SCID mice (PhoenixBio Co., Ltd., Higashihiroshima, Japan) via the spleen after butorphanol (Meiji Seika Pharma Co. Ltd., Tokyo, Japan) injection as previously described [14]. Three weeks after transplantation, 2 μL of blood was collected periodically from the tail vein to measure human albumin (h-Alb) concentration. Blood h-Alb levels in chimeric mice were measured by immunonephelometry in a model BM6050 autoanalyzer (JEOL, Tokyo, Japan) using LX Reagent Eiken Alb II (Eiken Chemical, Tokyo, Japan). All experimental animals reported in this article were housed with environmental enrichments under pathogen-free conditions and maintained in a 12-h light/dark cycle with sterilized ad libitum water and regular diet CRF-1 (Oriental Yeast, Tokyo, Japan). All experimental procedures were conducted in accordance with the guidelines provided by Proper Conduct of Animal Experiments (June 1, 2006; Science Council of Japan) and approved by the Animal Care and Use Committee of PhoenixBio Co., Ltd. (2287).

### Isolation and culture of cFHHs (PXB-cells^®^)

To isolate cFHHs, 14 to 17-week-old chimeric mice with blood h-Alb levels >12 mg/mL (estimated replacement indexes >85% [donor A through C; Table 1]) were used. cFHHs were isolated from 62 chimeric mice (9 mice for real-time quantitative reverse transcription polymerase chain reaction [qPCR] analysis, measurement of albumin secretion and CYP3A activity [Table 1], 6 mice for measurement of urea synthesis, 3 mice for CYP activity assay using a cocktail mixture of probe substrates, 6 mice for transporter immunohistochemistry and function assay, 24 mice for measurement of purity of h-hepatocytes, and 14 mice for culture under several cell density conditions and microarray analysis) which were euthanized by exsanguination from the inferior vena cava under anesthesia using isofluorane (Mylan Pharmaceuticals Inc., Canonsburg, PA) in a two-step collagenase perfusion method as described below.

**Table 1:** Data of isolated hepatocytes for evaluation of drug metabolizing enzymes (phase I and II) and transporters gene expression.

Donor	Animal No.	Sex	Age (weeks)	Body weight (g)	h-Alb concentration (mg/mL)	Yield of cells (cells/mouse)	Viability (%)
A BD195	A-1	male	15	18.79	17.2	2.24 × 10^8^	91.7
	A-2	male	15	17.42	16.4	1.69 × 10^8^	92.6
	A-3	male	15	19.06	17.1	1.52 × 10^8^	83.9
B BD342	B-1	male	16	17.45	12.6	1.31 × 10^8^	87.5
	B-2	male	16	17.54	15.2	1.53 × 10^8^	93.8
	B-3	male	17	17.19	12.6	1.42 × 10^8^	87.3
C BD85	C-1	female	14	15.3	13.0	1.42 × 10^8^	89.5
	C-2	male	15	14.4	14.0	1.27 × 10^8^	86.7
	C-3	male	15	20.6	14.3	1.04 × 10^8^	79.5

The liver was perfused at 38ºC for 10 min at 1.5 mL/min with Ca^2+^-free and Mg^2+^-free Hanks' balanced salt solution (CMF-HBSS) containing 200 μg/mL ethylene glycol tetraacetic acid (EGTA), 1 mg/mL glucose, 10 mM N-2 hydroxyethylpiperazine-N’-2- ethanesulfonic acid (HEPES), and 10 μg/mL gentamicin. The perfusion solution was then changed to CMF-HBSS containing 0.05% type IV collagenase (Sigma-Aldrich Japan, Tokyo, Japan), 0.6 mg/mL CaCl_2_, 10 mM HEPES, and 10 μg/mL gentamicin, and perfusion was continued for 9–12 min at 1.5 mL/min. The liver was dissected and transferred to a dish; liver cells were gently disaggregated in the dish with CMF-HBSS containing 1% bovine serum Alb, 10 mM HEPES, and 10 μg/mL gentamicin. The disaggregated cells were centrifuged three times (50× g, 2 min). The pellet was suspended in medium consisting of Dulbecco's modified Eagle's medium (DMEM), 10% fetal bovine serum (FBS), 20 mM HEPES, 44 mM NaHCO_3_, and antibiotics (100 IU/mL penicillin G and 100 μg/mL streptomycin). Cell number and viability were assessed using the trypan blue exclusion test.

For each assay, cFHHs isolated from a chimeric mouse or pooled cFHHs isolated from three or four chimeric mice were used. The cFHHs were inoculated in wells of type I collagen-coated 24-well or 96-well plates at 0.53, 1.06, 1.60, or 2.13 × 10^5^ cells/cm^2^, and cultured with dHCGM (DMEM containing 10% FBS, 2% dimethylsulfoxide [DMSO], 20 mM HEPES, 44 mM NaHCO_3_, 15 μg/mL L-proline, 0.25 μg/mL insulin, 5 × 10^-8^ M dexamethasone, 5 ng/mL epidermal growth factor, 0.1 mM L-ascorbic acid 2-phosphate, 100 IU/mL penicillin G, and 100 μg/mL streptomycin) as we previously reported [25, 26]. The medium was replaced every 3 to 4 days. Portions of cells from day 0 samples were used for CYP3A activity measurements (in suspension) and total RNA isolation. ch-hepatocytes of donor A were thawed and cultured as cFHHs.

### Measurement of purity of h-hepatocytes in cFHHs

Contamination by mouse cells in isolated cFHHs (donor A) was confirmed using 66Z rat IgG antibody specific for mouse cells [1]. The isolated cFHHs were incubated for 30 min with antibody conjugated to Dynabeads (Thermo Fisher Scientific, Waltham, MA) to recognize the surface of mouse cells, but not human cells. The ratio of bead-negative and -positive hepatocytes in the suspension of cFHHs was determined by microscopy. The cFHHs were inoculated on type I collagen-coated wells of 24-well plates (2.13 × 10^5^ cells/cm^2^) and cultured using dHCGM. The cells were fixed with 10% formaldehyde for 10 min and stained with 4 μg/mL Hoechst33258 (#S-23387; Sigma) for 10 min at 2, 13, 23, and 33 days at room temperature. The h-hepatocytes and m-cells were counted on the culture plates under a microscope. The h-hepatocytes were distinguished from mouse cells based on different patterns resulting from the Hoechst staining of the nuclei [27]. Two independent experiments were performed.

### Measurement of h-Alb concentration in culture supernatant and CYP3A activity

cFHHs (donor A, B, C, Table 1) were inoculated in wells of type I collagen-coated 24-well plates (2.13 × 10^5^ cells/cm^2^) and cultured with dHCGM for 21 days. Three wells were used for each condition. The culture supernatant was collected at 2, 7, 14, and 21 days, and h-Alb levels were measured by latex agglutination immunonephelometry as described above. Three independent experiments were performed using cFHHs isolated from three different mice (Table 1). The data are presented as mean ± SD.

Sample preparation for evaluation of the CYP3A activities of cFHHs was conducted as previously reported [1]. The isolated (day 0) or cultured hepatocytes at 2, 7, 14, and 21 days were incubated in Krebs–Henseleit buffer with 10 μM midazolam as substrate specific for the CYP3A subtype at 37°C for 2 h. The incubated solution was collected, and the concentration of the 1'-hydroxymidazolam metabolite was measured by high-performance liquid chromatography (HPLC) using a Lachome Elite device (Hitachi High-Technology Co., Tokyo, Japan). HPLC was performed at a flow rate of 1.0 mL/min using an Xterra RP18 column (4.6 × 150 mm, 5 μm; Waters). The measurements were performed in duplicate. Lower limit quantification of 1'-hydroxymidazolam was 0.1 μmol/L. Three independent experiments using cFHHs isolated from three different mice were performed (Table 1). The data are presented as mean ± SD.

### Measurement of mRNA expression levels by real-time qPCR

The cFHHs (donor A, B, C; Table 1) were inoculated in wells of type I collagen-coated 24-well plates (2.13 × 10^5^ cells/cm^2^) and cultured with dHCGM for 2, 7, 14, and 21 days. The mRNA expression levels of human and mouse genes were quantified by qPCR. Total RNA was isolated from each hepatocyte sample of three wells at the defined times using TRIzol (Invitrogen Corporation, Carlsbad, CA) and treated with DNase (Invitrogen). cDNA was synthesized using 1 μg of RNA, SuperScript III reverse transcriptase (Invitrogen), and random primers (Invitrogen) according to the manufacturer’s instructions, then subjected to qPCR. Genes were amplified with a set of gene-specific primers (S1 Table) and SYBR Green PCR mix (Applied Biosystems, Tokyo, Japan) using a 7500 Real-Time PCR System (Applied Biosystems). Cycling conditions were described previously [12]. Three independent experiments were performed using cFHHs isolated from three different mice (Table 1). The data are presented as mean ± SD. The species specificities of the human and mouse primers were confirmed using h- and m-hepatocyte cDNA, and non-specific amplification was not observed (data not shown).

### Immunostaining for MRP2

The cFHHs (donor A) were inoculated in wells of type I collagen-coated 96-well plates (2.13 × 10^5^ cells/cm^2^) and cultured with dHCGM for 2, 7, and 14 days. For indirect immunofluorescence analysis, the primary antibody was anti-MRP2 antibody (GeneTex, Inc. Irvine, CA). cFHHs were fixed with formalin for 10 min and permeabilized with 0.25% Triton X-100 in 10 mM PBS (pH 7.5) for 10 min at room temperature. After incubation in PBS containing 10% donkey serum for 30 min, cells were incubated with primary antibodies and diluted in PBS overnight at 4°C. After washing with PBS containing Tween, cells were incubated with Alexa 488-conjugated anti-mouse IgG donkey serum as the secondary antibody (Life Technologies Corporation, Carlsbad, CA) for 1 h at room temperature. Nuclei were stained with Hoechst 33258.

### Measurement of MRP2, sodium-taurocholate cotransporting polypeptide (NTCP), and BSEP activities

The cFHHs (donor A, pooled cFHHs from three or four animals) were inoculated in wells of type I collagen-coated 24-well plates (2.13 × 10^5^ cells/cm^2^) and cultured with dHCGM for 7 and 16 days (for the assay using CDFDA) or for 9 days (DBD). Sixteen days after plating, the cFHHs were incubated with 1.25 μM CDFDA, a substrate for MRP2, in Hanks’ Balanced Salt Solution (HBSS) for 5 min. The cells were washed twice followed by incubation with HBSS or Ca^2+^- and Mg^2+^-free HBSS. Cell morphology, bile canaliculi formation, and accumulation of 5-(and-6)-carboxy-2',7'-dichlorofluorescein (CDF) in bile canaliculi were analyzed with phase contrast and fluorescence microscopy (Bz-X, KEYENCE CORPORATION, Osaka, Japan), respectively. Seven days after plating, the cFHHs were preincubated in Ca^2+^- and Mg^2+^-free HBSS with or without cyclosporin A (CsA; 10 or 100 μM) for 30 min, followed by incubation with 1.25 μM CDFDA, with or without CsA (10 or 100 μM), for a further 20 min. Fluorescence of CDF was observed using fluorescence microscope (KEYENCE CORPORATION). Two independent experiments using cFHHs isolated from two different mice were performed to ensure reproducibility.

Nine days after plating, the cFHHs (donor A) were preincubated in HBSS with or without 10 μM CsA at 37°C for 30 min. This was followed by incubation with 10 μM DBD in the presence or absence of 10 μM CsA at 37°C for 10 min. The cFHHs were washed three times with HBSS at 4°C. The cells were incubated in HBSS at 37°C for 5 to 210 min. To inhibit substrate uptake by NTCP, cells were treated with CsA from the preincubation process onwards. To inhibit excretion of the substrate by the BSEP, cells were treated with CsA with the DBD treatment during the washing process. The fluorescence of DBD that had accumulated in bile canaliculi was analyzed using fluorescence microscope (KEYENCE CORPORATION). Two independent experiments using cFHHs isolated from two different mice were performed to ensure reproducibility.

### Microarray analysis

For microarray analysis, cFHHs (donor A, pooled cFHHs from 3-4 animals) were plated at high density (2.13 × 10^5^ cells/cm^2^) or low density (0.53 × 10^5^ cells/cm^2^) in wells of type I collagen-coated 24-well plates and cultured for 7 days. Total RNA was isolated from pooled cells of three wells of each fresh and cultured cFHH at high density or low density. RNA extraction was conducted as described above. RNA integrity was assessed using a Bioanalyzer (Agilent Technologies, Santa Clara, CA). After total RNA was deemed to be of sufficient quality (A260/A280 >1.9 and 28S/18S ratios approaching 2), the samples were stored at -80°C until further analysis. Three individual experiments using cFHHs isolated from three different mice were performed and these RNA samples were used for microarray analysis. For this analysis, RNA samples were applied to the GeneChip® Human Genome U-133 Plus 2.0 Array (Affymetrix, Santa Clara, CA) containing 54,675 probe sets according to the manufacturer’s instructions. Gene expression array data were normalized using the MAS5 algorithm (Affymetrix). The signal reliability of each probe was determined based on the MAS5 Call algorithm (Affymetrix), and each probe was assigned to one of three flags (P: present, M: marginal, and A: absent). To correct for bias between chips, GeneChip CEL files were imported into GeneSpring14.8 (Agilent Technologies). Correlation analyses for the RMA-normalized logarithmic expression levels of all probe sets were performed using GeneSpring14.8. We deposited our array data to NCBI GEO (Gene Expression Omnibus https://www.ncbi.nlm.nih.gov/geo/) (accession no. GSE153298).

### Statistical analysis

The data were analyzed with Statcel 4 (OMS Publishing Inc., Tokorozawa, Japan). Log_10_-transformed data obtained in qPCR analysis were used for statistical analysis. Results are expressed as mean ± SD, and the significance of the difference between two groups was analyzed by Student’s *t*-test when data were normally distributed, and otherwise by Welch’s *t*-test. Multiple-sample comparisons were made by one-way analysis of variance followed by post hoc analysis. Statistical significance was considered at p-values <0.05.

## Results

### Morphology of cFHHs and contamination of mouse cells in cFHHs

**Fig 1:**
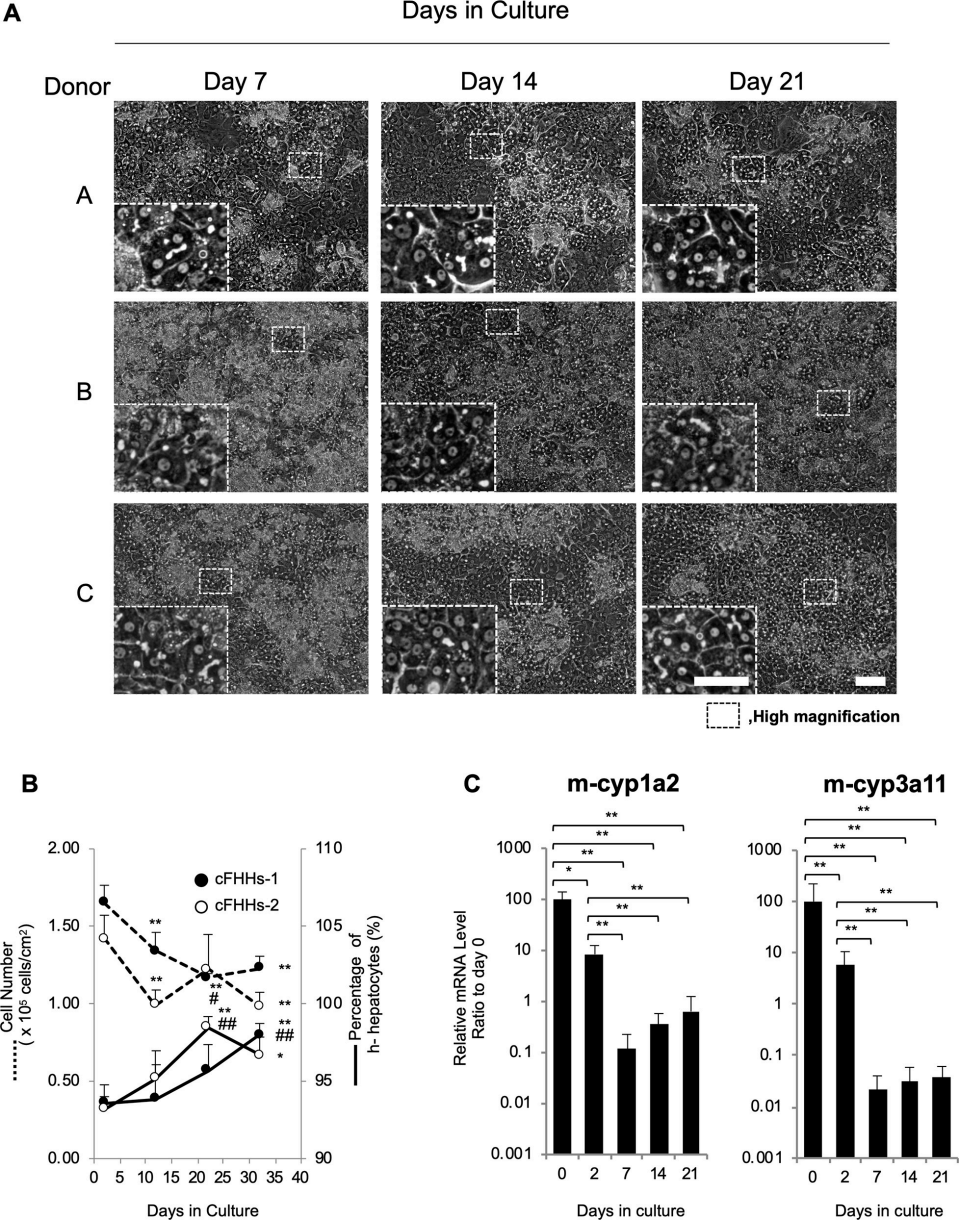
Morphology, viability, and purity of cFHHs during culture. **A:** Phase contrast microphotographs of cFHHs during culture at day 7, 14 and 21. cFHHs isolated from chimeric mice transplanted with three different donor cells (Donor A, B, and C) were plated at 2.13 x 10^5^ cells/cm^2^ and cultured. The inserts are high magnification images. Scale bars denote 100 μm in the low magnification images and 50 μm in the high magnification images. **B:** Cell numbers and ratio of h-hepatocytes during culture. cFHHs isolated from two different chimeric mice transplanted with donor A cells were plated (2.13×10^5^ cells/cm^2^) and fixed at days 2, 13, 23, and 33. After Hoechst staining, h-hepatocytes and mouse cells were counted by microscopic observation of three to five fields. Total cell numbers and percentages of h-hepatocytes are denoted by dotted and normal lines, respectively. **C:** Mouse gene expression levels during culture. Expression levels of m-cyp1a2 and 3a11 mRNA were measured by qPCR on days 0, 2, 7, 14, and 21. Data are expressed as mean ± SD. *p<0.05 versus at day 2; **p<0.01 versus at day 2; ^#^p<0.05 versus at day 12; ^##^p<0.01 versus at day 12.

To verify the lot-to-lot variations, cFHHs were isolated from chimeric mice transplanted with cells from the three different donors and cultured at high density. Animal information, yield, and viability of the cFHHs from each chimeric mouse are summarized in Table 1. At least 10^8^ cells were collected from each chimeric mouse. Regardless of the original characteristics of ch-hepatocytes (donor A and B: platable, donor C: non-platable) before transplantation into host mice, all cFHHs were highly platable. The cultured cFHHs showed matured hepatocyte morphology for at least 3 weeks and many bile canaliculi had formed between cells at 7 days (Fig 1A and S3 Fig). There were no clear differences in cFHH morphologies among the donors (Fig 1A).

To analyze the presence of contaminating mouse cells, freshly isolated cFHHs were treated with 66Z-conjugated magnetic beads. In this culture condition, hepatocytes do not proliferate. Microscopic observation revealed that the average ratio of h-hepatocytes to total cells in the freshly isolated cFHHs was 90.3 ± 2.9% (22 animals). We also counted the number of h-hepatocytes and contaminating mouse cells at 2, 13, 23, and 33 days after plating. Two days after plating, the cultured cell density (h-hepatocytes and mouse cells) was approximately 1.5 × 10^5^ cells/cm^2^. At day 13, cell density decreased to approximately 80% of the day 2 density, and then remained stable for at least 33 days. The m-hepatocytes decreased in this condition, and the ratio of h-hepatocytes to total cells was 93% at 2 days, and then gradually increased to 98% at 33 days (Fig 1B). These results suggested that the present culture conditions were more suitable for h-hepatocytes than for mouse cells.

The expression profiles of mouse cyp (m-cyp) 1a2 and 3a11 mRNAs were analyzed by qPCR using mouse-specific primer sets. Expression of both m-cyp mRNAs drastically decreased and the lowest level was <1% (cyp1a2) and 0.1% (cyp3a11) of basal expression level (day 0) at 7 days (Fig 1C).

### Evaluation of h-Alb secretion, urea synthesis, CYP activities, and CYP, UGT, and transporter mRNA expressions in cFHHs

**Fig 2:**
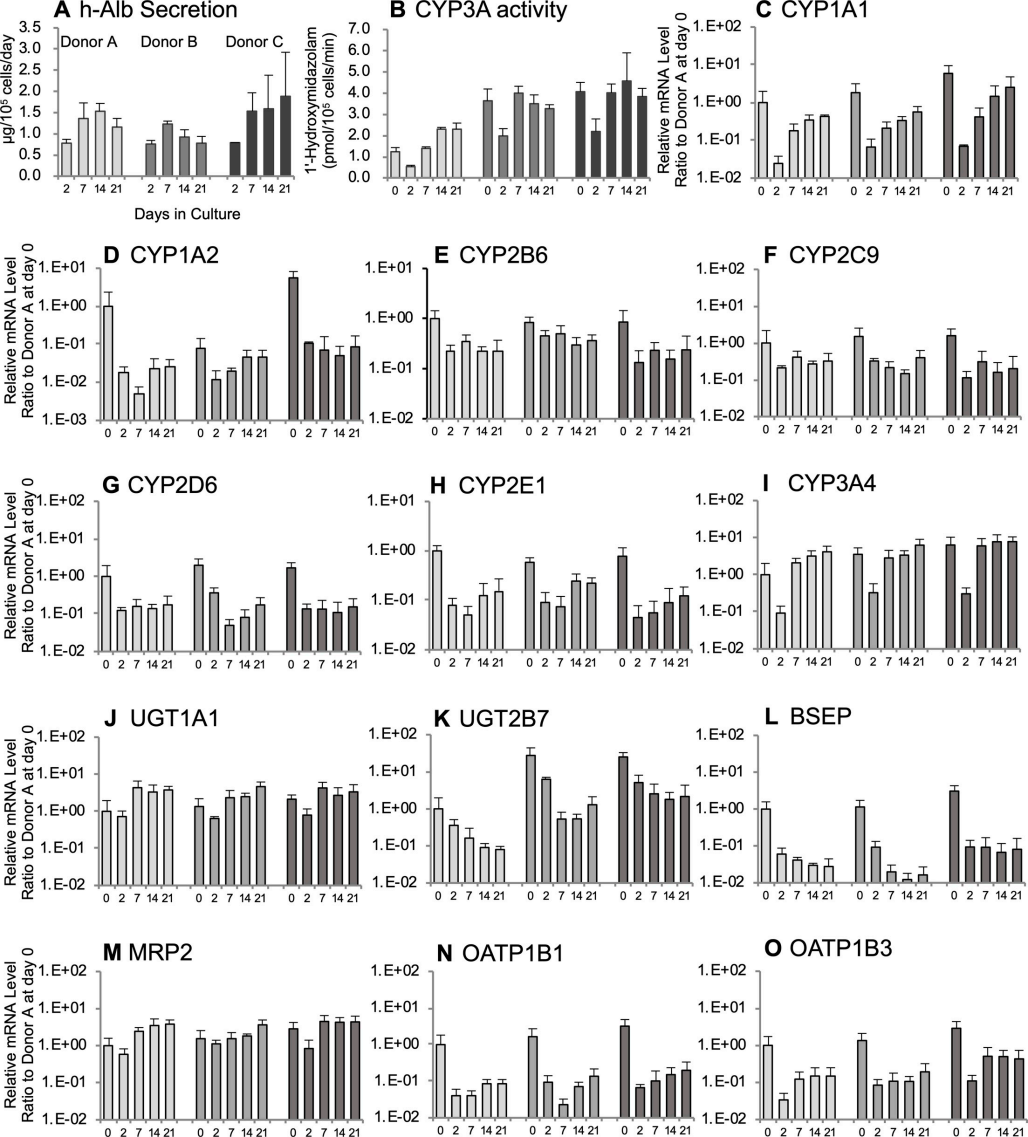
Hepatic function and gene expression in cFHHs during culture. **A:** h-Alb production, **B:** CYP3A activities, **C-O:** Human gene expression levels (C: CYP1A1, D: CYP1A2, E: CYP2B6, F: CYP2C9, G: CYP2D6, H: CYP2E1, I: CYP3A4, J: UGT1A1, K: UGT2B7, L: BSEP, M: MRP2, N: OATP1B1, O: OATP1B3). cFHHs collected from chimeric mice transplanted with cells of three different donors (A, B, and C) were plated at 2.13×10^5^ cells/cm^2^ and cultured for up to 21 days. Results represent means ± SD of three independent experiments. The Y-axis of C to O represents the relative expression level of each gene to that in freshly isolated cFHHs originating from donor A. Day 0 shows the values of isolated cFHHs.

All cFHHs showed similar h-Alb secretion ability at 2 days (approximately 0.7 μg/10^5^ cells/day) and 7 days (approximately 1.4 μg/10^5^ cells/day). After day 7, the secretion of albumin was maintained until day 21 (Donor A and C) or gradually reduced to day 2 levels (Donor B) (Fig 2A).

The synthesis of urea by cFHHs (donor A) was measured at days 8 and 15. Stable urea synthesis levels were observed in cFHHs at days 8 (1.47 mM/day, 110.42 μg/10^6^ cells/day) and 15 (1.42 mM/day, 106.44 μg/10^6^ cells/day). These data were similar to those in the previous report (0.42 mM/day, approximately 113.28 μg/10^6^ cells/day) [28].

The CYP3A activity of cFHHs was measured using midazolam as a substrate (Fig 2B). The activities in suspension (day 0) were different among the donors, but all activities decreased at 2 days and then recovered to or increased over basal levels after 7 days. The CYP3A activity in donor A was lower than in those in donors B and C. The recovered activity levels were maintained during the culture period. According to the mouse hepatocyte contamination rate and m-cyp3a11 mRNA expression levels (Fig 1B and 1C), CYP3A activities in cFHHs might be derived from human hepatocytes.

Expression patterns of several human hepatic genes were analyzed by qPCR using cultured cFHHs from three donors (Fig 2C–2O). Two genes, CYP1A2 and UGT2B7, showed donor differences in these expression levels at day 0. In the case of cFHHs, the expression level of CYP1A2 in donor B was 10 times lower than in donors A or C (Fig 2D). The expression level of UGT2B7 in donor A was also 10 times lower than those in donors B and C in the case of cFHHs (Fig 2K). Except for CYP1A2 and UGT2B7, the other hepatic genes showed similar basal expression levels at day 0. Through the culture period, the h-hepatocytes from the three donors showed similar expression profiles. Two expression patterns were evident during culture. In the first pattern, expression levels decreased at day 2 and then remained stable between 2% and 120% of the initial levels (day 0, isolated hepatocytes; CYP1A1, CYP1A2, CYP2B6, CYP2C9, CYP2D6, CYP2E1, UGT2B7, BSEP, organic anion transporting polypeptide [OATP] 1B1, and OATP1B3). In the second pattern, expression levels decreased at 2 days, but increased to higher than the initial levels, which were maintained until 21 days (CYP3A4, UGT1A1, and MRP2). The expression pattern of CYP3A4 mRNA was very similar to CYP3A activity (Fig 2B and 2I). Although CYP3A activities of donor A was a little lower than those of donor B and C, mRNA expressions of CYP3A4 were similar among the three donors. The mRNA expression levels of 10 of 13 CYP and UGT enzymes and transporters were maintained at >10% of the levels of freshly isolated cFHHs after 21 days. Those of hUGT2B7 (7.1%), hBSEP (2.3%), and hOATP1B1 (7.7%) were decreased by <10% of the initial levels (Table 2).

**Table 2:** Relative gene expression levels of human drug metabolizing enzymes (phase I and II) and transporters.

	Days in culture											
	2			7			14			21		
**Phase I enzymes**												
hCYP1A1	2.4	±	1.2	12.2	±	5.5	25.9	±	8.2	38.9	±	7.0
hCYP1A2	6.3	±	7.6	9.0	±	14.1	20.7	±	33.1	21.0	±	32.8
hCYP2B6	29.7	±	21.5	40.3	±	16.9	25.1	±	9.2	31.1	±	11.0
hCYP2C9	16.6	±	8.2	25.0	±	14.3	15.6	±	9.8	23.8	±	10.0
hCYP2E1	9.7	±	5.2	8.4	±	4.1	21.4	±	17.1	23.2	±	12.1
hCYP2D6	13.0	±	5.2	8.8	±	6.7	8.2	±	5.1	11.8	±	4.9
hCYP3A4	7.8	±	2.5	127.8	±	70.6	178.7	±	122.1	238.3	±	155.1
**Phase II enzymes**												
hUGT1A1	52.4	±	17.9	269.2	±	143.2	214.2	±	105.4	292.9	±	118.2
hUGT2B7	26.3	±	8.4	9.4	±	7.2	6.1	±	3.7	7.1	±	2.1
**Transporters**												
hBSEP	5.7	±	2.5	3.0	±	1.2	2.1	±	1.0	2.3	±	0.7
hMRP2	53.5	±	22.0	167.4	±	72.5	206.1	±	125.3	256.8	±	114.8
hOATP1B1	3.9	±	1.8	2.9	±	1.3	5.9	±	2.3	7.7	±	1.3
hOATP1B3	4.5	±	1.6	12.6	±	4.8	13.3	±	4.9	14.7	±	0.3

Expression levels of the genes in PXB-cells cultured for 2, 7, 14, and 21 days were compared to those in freshly isolated PXB-cells (day 0). The results represent mean (ratio (%) of the day 0 level) ± SD of three independent experiments.

### Comparison of characteristics between cFHHs and ch-hepatocytes from same donor or fresh adult h-hepatocytes

The ch-hepatocytes from donor A were specified by the provider as platable hepatocytes with CYP induction and transporter abilities. The ch-hepatocytes were thawed and cultured in the same conditions as cFHHs for 14 days to compare their morphologies, h-Alb secretion, and CYP and UGT mRNA expression levels. In the present culture conditions, the ch-hepatocytes also maintained matured hepatocyte morphology. The results of h-Alb quantification indicated that there was no significant difference in the h-Alb production abilities of the cultured ch-hepatocytes and the cFHHs after at least 14 days (S1 Fig). qPCR analysis revealed that there was a difference of over two log in the CYP3A4 mRNA expression levels between the ch-hepatocytes and cFHHs at day 0. However, after day 2, the CYP3A4 mRNA expression levels in the cultured ch-hepatocytes were comparable or slightly lower than those in the cultured cFHHs at the same time points. The mRNA expression patterns of the CYPs and UGTs were similar to those in cFHHs (S1 Fig). The ch-hepatocytes of donor C did not attach to the dishes as observed in donor A.

The cryopreserved hepatocytes used for transplantation in chimeric mice were derived from very young donors (2-5 years old). To confirm the phenotype of hepatic expression in the chimeric mouse liver, we compared hepatic gene expression (CYPs and UGTs) in cFHHs (from donor A, B, and C) and fresh h-hepatocytes from 4 adult donors (25-, 39-, 57-, and 61-year-old). The expression levels of CYP1A2 and UGT1A1 among the measured 6 genes (CYP1A2, CYP2C9, CYP2D6, CYP3A4, UGT1A1, and UGT2B7) in cFHHs were similar to those in h-hepatocytes from adult donors. Although the expression levels of CYP2D6 and CYP3A4 were higher and those of UGT2B7 were lower in fresh h-hepatocytes than in cFHHs, there were no statistical differences between them (S2 Fig).

### Localization and function of MRP2, NTCP, and BSEP in cFHHs

**Fig 3:**
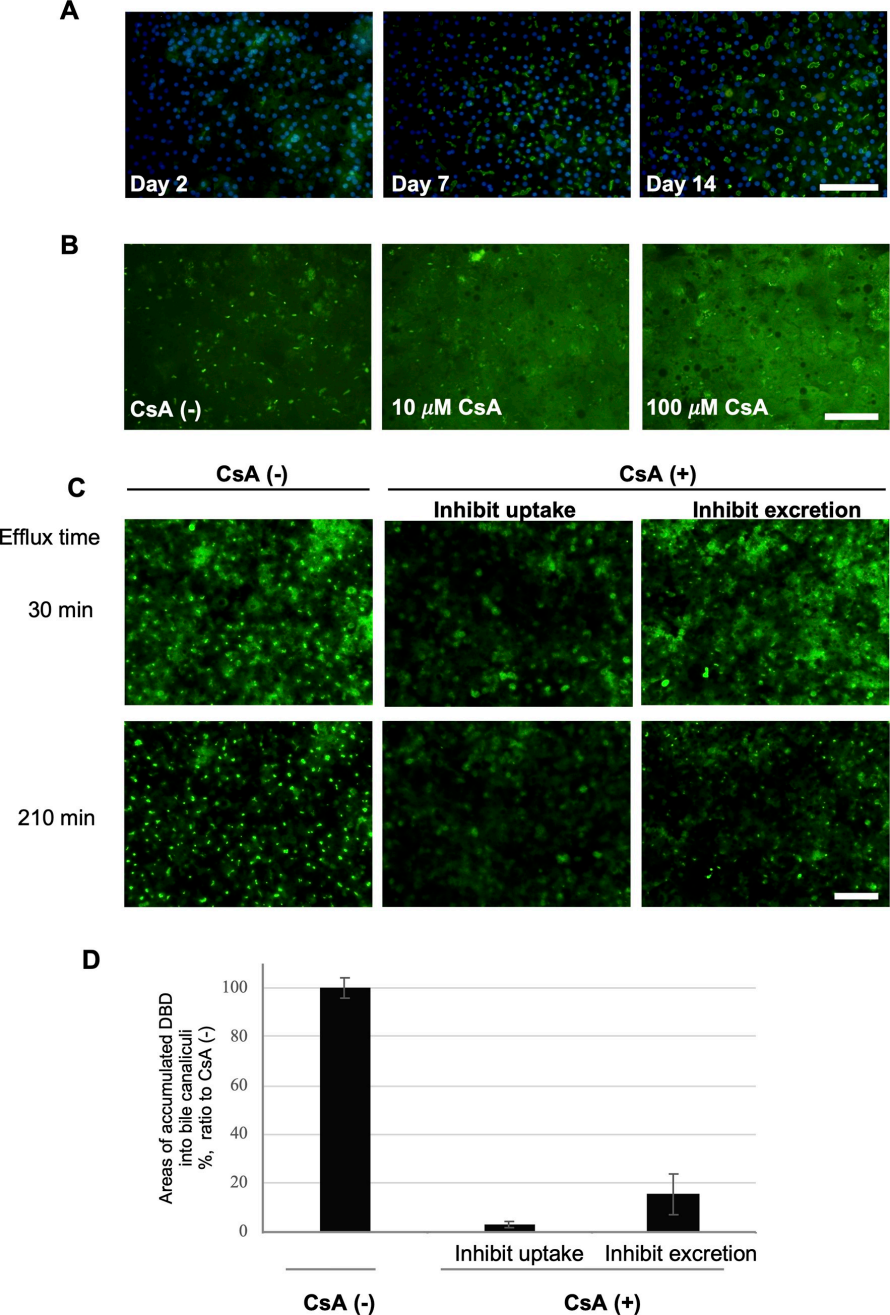
Expression and function of transporters in cFHHs during culture. **A**: Transporter protein localization and activity in cultured cFHHs. cFHHs (donor A) were plated at 2.13 × 10^5^ cells/cm^2^. cFHHs were cultured for 7 and 14 days and fixed, and MRP2 protein expression was determined by immunostaining. Images of cFHHs after double staining with MRP2 (green) and Hoechst (blue) are presented. Scale bar denotes 100 μm. **B**: MRP2 transporter activity was determined in cultured cFHHs using CDFDA. cFHHs were incubated with CDFDA and CsA (10 or 100 μM) at day 7. CsA treatment inhibited the accumulation of CDF in the bile canaliculi, and intense fluorescence signals were observed in the cytoplasm at 10 and 100 μM CsA. Scale bar denotes 100 μm. **C**: NTCP and BSEP transporter activities were determined in cultured cFHHs using DBD and CsA. DBD was incorporated into cFHHs and accumulated in bile canaliculi between cFHHs at day 9, as shown in green. To inhibit uptake of the substrate by NTCP, cells were treated with CsA from the preincubation process onwards, before DBD treatment. To inhibit excretion of the substrate by BSEP, cells were treated with CsA from the washing process onwards, after DBD treatment. Scale bar denotes 100 μm. Effect of CsA treatment on DBD-uptake in cells via NTCP, and DBD-excretion in bile canaliculi via BSEP. **D**: Photographs of microscopy images were taken at 210 min and areas of DBD accumulation in bile canaliculi were measured using a Bz-X analyzer. DBD that had accumulated in bile canaliculi was expressed as a ratio with the control (CsA [–]).

Localization of the efflux transporter MRP2 in the cFHHs of donor A was confirmed by immunocytochemistry. There were no obvious signals in the cFHHs at day 0 and 2 (Fig 3A). However, MRP2 was localized to bile canalicular membranes at 7 days and the positive spots were enlarged at 14 days (Fig 3A). To analyze the transporter activity of MRP2, the cultured cFHHs (donor A) were treated with CDFDA at 16 days. Sustained accumulation of CDF, a fluorescent metabolite of CDFDA, was observed in the bile canaliculi in the presence of Ca^2+^ (S3 Fig). On the other hand, the CDF signals rapidly disappeared from the bile canaliculi in the absence of Ca^2+^. This observation indicated that in the presence of Ca^2+^ the integrity of the bile canalicular networks remains intact, while in the absence of Ca2^+^ the integrity of the canalicular space is disrupted, causing leakage of the canalicular contents, as reported previously [29]. Treatment with the potent MRP2 inhibitor CsA at 10 and 100 μM significantly inhibited the accumulation of CDF in the bile canaliculi, and intense fluorescence signals were observed in the cytoplasm (Fig 3B). To analyze the transporter activity of NTCP and BSEP, the cultured cFHHs (donor A) were treated with DBD at 9 days. DBD was incorporated into the cFHHs and accumulated in bile canaliculi (Fig 3C). To inhibit uptake of the substrate by NTCP, cells were treated with CsA from the preincubation process onwards, before treatment with DBD at 37°C. To inhibit excretion of the substrate by BSEP, cells were treated with CsA from the washing process onwards, following DBD treatment at 37°C. Treatment with CsA before or after DBD addition decreased the accumulation of DBD in the bile canaliculi (Fig 3C and 3D). cFHHs retained MRP2 and BSEP activities, although mRNA expression level of BSEP were 2% to 3% at 7, 14, and 21 days compared with day 0 (Table 2).

### Effects of cell density on morphologies and liver-specific functions of the cultured cFHHs

**Fig 4:**
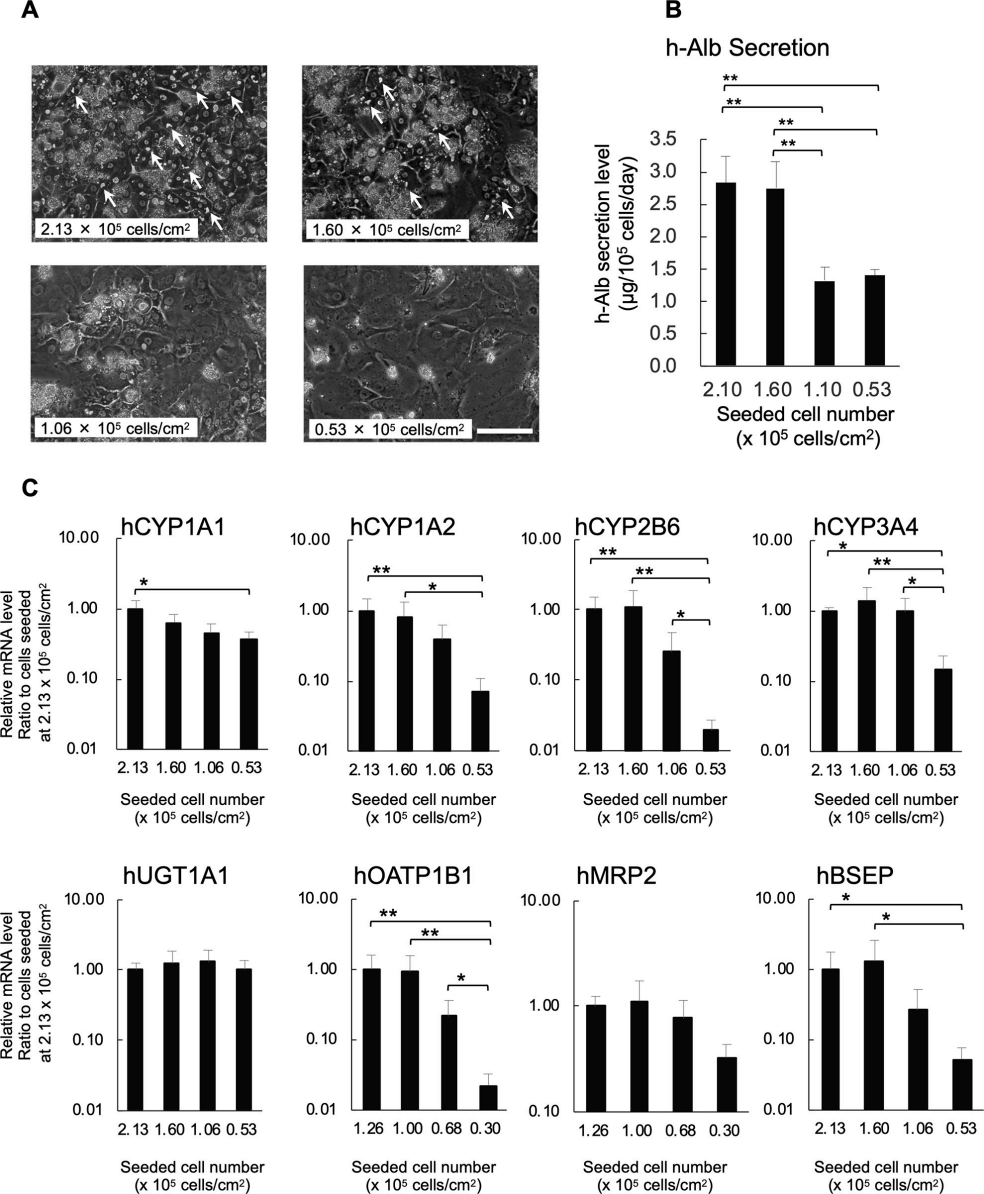
cFHHs cultured at different cell densities. **A:** Phase contrast microphotographs of cFHHs plated at 0.53, 1.06, 1.60, and 2.13×10^5^ cells/cm^2^ and cultured for 7 days. Arrows show bile canalicular structures. Scale bar denotes 100 μm. **B:** h-Alb levels in culture supernatant of cFHHs cultured at different cell densities. These results represent the mean ± SD of three independent experiments. **p<0.01 C: Gene expression levels of human phase I and II enzymes in cFHHs plated at 0.53, 1.06, 1.60, and 2.13 × 10^5^ cells/cm^2^ and cultured for 7 days. Each gene expression level was measured by qPCR. These results represent the mean ± SD of three independent experiments. *p < 0.05, **p < 0.01.

We predicted that the high density culture might induce the maintenance of high gene expression levels of phase I and II enzymes and transporters. To test this hypothesis, cFHHs were inoculated on type I collagen-coated plates at densities of 0.53, 1.06, 1.60, and 2.13 × 10^5^ cells/cm^2^. The freshly isolated cFHHs were highly platable, and firmly attached to type I collagen-coated plates 15 to 30 min after plating. Seven days after plating, cells inoculated at 0.53 and 1.06×10^5^ cells/cm^2^ were pre-confluent, and cells and nuclei were flattened at these plating densities (Fig 4A). When hepatocytes were plated at 1.60 and 2.13 × 10^5^ cells/cm^2^, they became confluent. Cells cultured at 2.13 × 10^5^ cells/cm^2^ were densely packed and bile canalicular structures were clearly observed between hepatocytes. However, cells plated at 1.60 × 10^5^ cells/cm^2^ were somewhat flat and bile canalicular structures were fewer than those between cells plated at 2.13 × 10^5^ cells/cm^2^ (Fig 4A). Microscopic observation revealed that approximately 60% to 73% of cells were attached to the plate surface in the various density conditions at 7 days (Table 3). To evaluate the effect of cell density on hepatocyte functions, h-Alb levels in the culture media were examined at 7 days. The h-Alb levels per 10^5^ attached cells at 7 days are shown in Fig 4B. Compared with those cultured at lower cell densities, cFHHs cultured at higher densities displayed significantly higher albumin secretion (Fig 4B). The qPCR results clearly indicated that the mRNA expression levels of CYP1A1, CYP1A2, CYP2B6, CYP3A4, MRP2, OATP 1B1, and BSEP decreased in the PXB-cells cultured at the lowest density, although the difference in MRP2 expression was not statistically significant (Fig 4C). However, UGT1A1 expression was stable at all examined densities

**Table 3:** Attached cell number and ratio of the number of attached cells to seeded cell number.

Seeding cell number (× 10^5^ cells/cm^2^)	Attached cell number on day 7 (× 10^5^ cells/cm^2^)			Cell adhesion ratio on day 7 (%)		
2.13	1.27	±	0.22	59.75	±	10.27
1.60	1.00	±	0.18	62.36	±	11.30
1.06	0.74	±	0.08	69.42	±	7.91
0.53	0.39	±	0.02	72.51	±	3.53

From these results, gene expression profiles of isolated cFHHs and cultured cFHHs from donor A at high density (2.13 × 10^5^ cells/cm^2^) or low density (0.53 × 10^5^ cells/cm^2^) were compared by microarray analysis. The gene expression profile of cFHHs cultured at 7 days at high density was close to that of isolated cFHHs (correlation efficiency: 0.911). In contrast, correlation efficiency between isolated cFHHs and cFHHs cultured at low density at 7 days was very low (0.736).

The mRNA expression profiles of cFHHs cultured at high density and low density were compared. Gene profiles were compiled using microarrays representing 54675 human transcripts. Among these, 22893 transcripts (42% of total probes) were assigned as present (P flag) for all cFHHs at either high density or low density. Moreover, of those 22893 transcripts, 1247 (5.4%) were expressed at levels two-fold lower in the cFHHs at low density than at high density (p<0.05, two-sided Student’s *t*-test).

Pathway analysis was then performed on the 1247 transcripts down-regulated in cFHHs at low density on day 7. As a result, 32 pathways were detected (p>0.001 minimum number of matches > 10), of which five were nuclear receptor (NR)-related pathways (Table 4). The 1247 transcripts down-regulated in low density cultured cFHHs included seven transcription factors (TFs), 27 phase I metabolic enzymes, 11 phase II metabolic enzymes, and 53 transporters. Down-regulated TF, phase I and II enzymes, and transporter genes are shown in S2 Table.

**Table 4:** Top 20 pathways in descending order in pathway analysis (p>0.001 minimal number of matches>10) on day 7.

Pathway	p-value
**Hs_Constitutive_Androstane_Receptor_Pathway_WP2875_94791**	0
Hs_Statin_Pathway_WP430_90508	0
Hs_Blood_Clotting_Cascade_WP272_93044	0
**Hs_Nuclear_Receptors_in_Lipid_Metabolism_and_Toxicity_WP299_89331**	0
**Hs_Pregnane_X_Receptor_pathway_WP2876_94792**	0
Hs_Tamoxifen_metabolism_WP691_92400	6.34E-19
Hs_Fatty_Acid_Omega_Oxidation_WP206_94194	1.76E-15
**Hs_PPAR_Alpha_Pathway_WP2878_94794**	6.16E-15
Hs_Drug_Induction_of_Bile_Acid_Pathway_WP2289_88593	1.53E-14
Hs_Fatty_Acid_Biosynthesis_WP357_94197	1.73E-14
Hs_Fatty_Acid_Beta_Oxidation_WP143_94768	2.96E-11
Hs_Tryptophan_metabolism_WP465_94086	3.22E-11
Hs_Amino_Acid_metabolism_WP3925_90737	4.11E-11
Hs_Complement_and_Coagulation_Cascades_WP558_90196	4.64E-11
Hs_One_carbon_metabolism_and_related_pathways_WP3940_94312	6.82E-11
**Hs_PPAR_signaling_pathway_WP3942_94205**	9.45E-11
Hs_Oxidation_by_Cytochrome_P450_WP43_94176	9.88E-11
Hs_Vitamin_B12_Metabolism_WP1533_85340	1.03E-10
Hs_Selenium_Micronutrient_Network_WP15_94185	1.19E-10
Hs_Metapathway_biotransformation_WP702_73516	4.19E-10

### mRNA expression of HNF4α and other TFs in cFHHs

**Fig 5:**
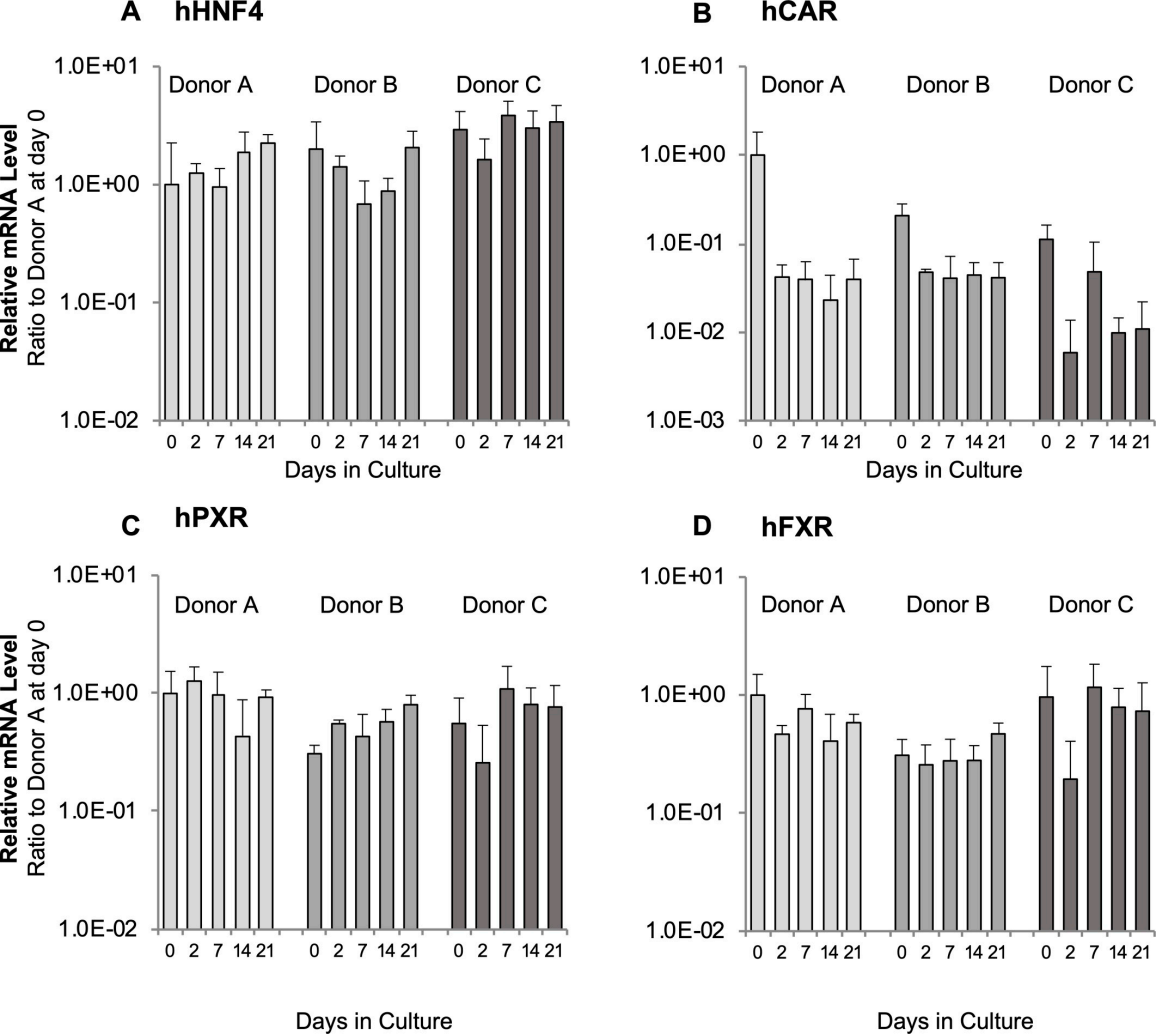
Human TF gene expression levels in cultured cFHHs. **A:** hHNF4α, **B:** hCAR, **C:** hPXR, **D:** hFXR. cFHHs from three different donors were plated at 2.13 × 10^5^ cells/cm^2^ and cultured for up to 21 days. The gene expression levels were measured by qPCR. Results indicate the mean ± SD of three independent experiments. The y-axis represents the relative expression level of each gene to the level in freshly isolated cFHHs originating from donor A. Day 0 shows the mRNA levels of isolated cFHHs.

In NR-related pathways detected by pathway analysis, the gene expression levels of significantly down-regulated TFs including NRs (HNF4α, CAR, and PXR) and an unchanged NR (FXR) were determined from the cFHHs of three donors (Table 1) cultured for 21 days. The mRNA expression of HNF4α, PXR, and FXR were quantified using qPCR. Levels were maintained at day 0 levels for at least 21 days. In contrast, CAR expression decreased to approximately 10% at day 2 and this level was maintained for 21 days (Fig 5). From these results, we concluded that CYP and UGT genes and transporter genes might be regulated by the expression of these TFs in high density cultured cFHHs.

## Discussion

It is recognized that ch-hepatocytes are the gold standard for *in vitro* studies of DMPK and toxicology [30–33]. However, the use of ch-hepatocytes is limited by lot size (at most several hundred vials from one donor), and there are individual differences among donors. Because of these limitations, researchers have been using immortalized liver-derived cell lines, such as HepG2, HepaRG, and induced pluripotent stem cell-derived hepatocytes for their *in vitro* studies [34]. Although some liver-specific functions are confirmed in these cells, many other liver functions were still significantly lower than those in primary h-hepatocytes [34–36].

In the case of humanized-liver mice, the transplanted ch-hepatocytes dramatically proliferate more than 1000-fold in the host mouse liver. Therefore, more than 10^8^ hepatocytes can be isolated from a chimeric mouse. Our previous results revealed that most genes showed comparable expression levels in the human hepatocytes of both human and chimeric mouse livers [12]. The humanized-liver mouse has been used in many studies of DMPK, safety, and hepatitis B virus and hepatitis C virus infections. These facts indicate that chimeric mice transplanted with ch-hepatocytes derived from one donor could stably and repeatedly supply a large amount of fresh h-hepatocytes.

In general, h-hepatocytes undergo changes in cell morphology and rapidly lose their hepatic functions, such as CYP expression and albumin secretion, in tradittional 2D culture model [37, 38]. To overcome this problem, several different types of culture methods have been established [6, 39]. In this study, we developed a novel culture system in which human hepatocytes maintain high hepatic function for at least 21 days. In this culture system, cFHHs isolated from PXB-mice were plated on type I collagen-coated plates at high density (2.13 × 10^5^ cells/cm^2^) in the dHCGM medium we previously developed [25]. h-Alb secretion, CYP3A activity, and mRNA expression levels were compared among three types of cFHHs isolated from three PXB-mice transplanted using different donors. Individual differences between mice were small in all data tested. There were no significant differences between the hepatic function of donors, except for CYP3A activity and the mRNA expressions of hCYP1A2 and hUGT2B7.

We previously compared the activities of cFHHs and the corresponding cryopreserved ch-hepatocytes [1]. As a result, the CYP1A2, 2C19, 2D6, and 3A activities in cFHHs were more than twice those in the cryopreserved ch-hepatocytes. We also compared the activities in cFHHs and cryopreserved cFHHs by a programmed freezer. Freezing decreased the CYP1A2 and 2D6 activities significantly. The decreased metabolism in cryopreserved hepatocytes may be attributable to two mechanisms: inactivation of P450 enzymes and loss of the cofactor NADPH due to cell membrane damage [40]. In addition, the CYP activities of cFHHs and thawed ch-hepatocytes (manufacture’s data, donor D) were compared. The activity of CYP1A2 using phenacetin in cFHHs and thawed ch-hepatocytes was 75.9 ± 1.8 and 3.9 pmol/10^6^ cells/min, respectively. The activity of CYP2C19 using S-mephenytoin in cFHHs and thawed ch-hepatocytes was 35.5 ± 0.9 and 3.1 pmol/10^6^ cells/min, respectively. The activity of CYP3A using midazolam in cFHHs and thawed ch-hepatocytes was 18.4 ± 1.2 and 5.0 pmol/10^6^ cells/min, respectively. These results of the CYP activities of cFHHs were similar or higher than those in ch-hepatocytes. We measured the major CYPs and UGTs mRNA expression levels in the ch-hepatocytes of donor A, resulting in extremely low levels (1/5-1/1000 compared to cFHHs), most likely due to the degradation of RNAs as a result of freeze and thaw. Interestingly, after 2 days, the mRNA expression levels of ch-hepatocytes recovered to levels of cFHHs (S1 Fig).

Several previous studies have reported that cultured cell density could affect hepatocyte-specific functions in h-hepatocytes [41], rat hepatocytes [42], and HepaRG [43]. Regardless of the culture models and hepatocyte sources, the cell density of cultured hepatocytes might be one of the most essential factors for the maintenance of liver-specific functions *in vitro*. We predicted that the high density culture of cFHHs should generate long-term high liver function levels. To test this hypothesis, cFHHs were cultured at both high density and low density, and the gene expression levels were compared using microarray analysis. The mRNA expressions of 1247 genes, including phase I and II metabolic enzymes and transporter genes, were significantly up-regulated in the high density culture conditions compared to low density conditions. NR pathways were significantly overrepresented following pathway analysis of the 1247 genes. The mRNA expression levels of HNF4α and six TFs were up-regulated in high density culture. The mRNA expression of HNF4α, PXR, and FXR in high density culture at 21 days were maintained at similar levels to the freshly isolated cFHHs, while the level of CAR mRNA decreased to approximately 10% of the level in freshly isolated cFHHs.

HNF4α and the CAR, PXR, and FXR control hepatic genes including CYP, UGT, and transporters. CYP2B6, 2C9, 2D6, and 3A4 are regulated by HNF4α [44–52]. The expression of CYP2B6 and CYP2C9 mRNA are also affected by CAR and PXR [53–55]. Although, the expression of PXR and HNF4 in cultured cFHHs were maintained at day 0 levels for 21 days, a CAR expression decreased to approximately 10% of day 0 level during the culture period. We suggest that the decreased expressions of CYP2B6 and CYP2C9 in cultured cFHHs might be due to decreased CAR expression. OATP1B1 is regulated by PXR, FXR, and CAR [56–59], suggesting that its gene expression in cultured cFHHs might be affected by the decrease in CAR expression.

CAR may also regulate expressions of CYP3A4, MRP2, and UGT1A1 [60–65]. However, their gene expressions were maintained in cFHHs in the present study, suggesting that other regulatory mechanisms might be involved in their gene expressions. In addition to HNF4α, PXR, CAR and FXR, CYP1A1 and UGT1A1 are affected by AhR. It was reported that C/EPBa and VDR affect CYP2D6 and MRP2, respectively. These data highlight the need for further explorations of the changes in the expression of these genes.

In this study, we provide the first evidence that the mRNA expressions of HNF4α, PXR, and FXR are maintained in high density culture conditions. These results suggest that the expression levels of some TFs might affect the maintenance of hepatic genes, such as CYP UGT, and transporters of cFHHs in high density culture conditions. Better understanding of the cross-talk mechanisms of TFs and their target genes, such as CYPs and UGTs, and transporters *in vitro* and *in vivo* by optimization of culture conditions are important. Further experiments involving siRNAs or gene transfer-mediated overexpression of these TFs into cFHHs are needed.

In this paper, we mainly measured the mRNA expression levels of metabolic enzymes, and the CYP3A activity, albumin secretion, and urea synthesis were determined during culture. Recently, we published the CYP activities of cFHHs of donor A after 21 days in the same culture condition [66]. CYP mRNA expression in the present study was compared with the corresponding activities in this paper during culture. The mRNA expression levels and the activities of CYP1A2 and CYP2D6 were decreased at day 7 and remained at similarly low levels until day 21. On the other hand, the CYP2C9 mRNA decreased gradually and reached 0.3 at day 21 compared to that at day 0; however, the activity was around 1.0 during culture. The CYP3A4 mRNA levels increased gradually and reached 4.0 at day 21 compared to that at day 0; however, the activity of CYP3A was around 1.0 during culture. Thus, the relation of the mRNA expression levels and activities were different among CYPs.

We already compared the CYP activities of cFHHs derived from young donors and adult PHHs (54-, 57-, and 75-year-old) in our previous paper [1]. The resulting CYP activities were similar between the cFHHS from young donors and adult PHHs. We have newly determined the level of mRNA CYP expression between cFHHs from young donors and adult PHHs, resulting there were no statistical differences between them (S2 Fig)

A disadvantage to the use of humanized-liver mice as a source of h-hepatocytes is contamination by mouse parenchymal and non-parenchymal cells. In this study, h-hepatocytes isolated from chimeric mice showed a high replacement index (>90%). In a previous study, we demonstrated that contamination with m-hepatocytes did not show any effects on the results of a drug metabolizing assay using cFHHs in suspension cultures [1]. In this study, we investigated the viability of the mouse cells and several liver-specific gene expressions of m-hepatocytes in the culture conditions. The ratio of mouse cells to total cultured cells was approximately 6% to 7% at 2 days after seeding and decreased to 2% to 3% at 32 days. In addition, m-cyp1a2 and cyp3a11 mRNAs drastically and quickly decreased to 0.1% to 1% of the basal expression levels during the culture period. These results suggest that the m-cyps, at least cyp1a2 and 3a11, only marginally, if at all, affect the results of the metabolic study using the present *in vitro* model.

We compared the hepatic functions of cFHHs and original ch-hepatocytes in the present culture conditions. The original ch-hepatocytes (donor A) are commercially as Transporter-Qualified Plateable Cryopreserved Human Hepatocytes. The original hepatocytes also attached on collagen-coated plates at high cell densities, as did the cFHHs, and the expression levels of CYP3A4 mRNA in the original ch-hepatocytes were comparable or lower than those in the cFHHs at 7 days and 14 days. On the other hand, the original ch-hepatocytes derived from donor C showed poor platability, and it was impossible for them to attach in the culture conditions even at high density (data not shown). These results suggest that, regardless of the characteristics of the original ch-hepatocytes, the liver-specific functions of cFHHs were comparable or higher than those of ch-hepatocytes classified as the highest grade.

Here, we demonstrated that cFHHs cultured at a high density could stably maintain several hepatic functions for long periods, using a conventional culture method with collagen-coated plates. In conclusion, cFHHs are a valuable tool for *in vitro* metabolic and pharmaco-toxicological studies.

## Supporting information

S1 File (DOCX)

S1 Fig h-Alb levels and gene expression levels of human phase I and II enzymes in cultured PXB-cells and original ch-hepatocytes (donor A). PXB-cells originating from donor A and original ch-hepatocytes (donor A) were plated at 2.13 × 10^5^ cells/cm^2^ and cultured for up to 14 days. h-Alb levels in the culture supernatant were measured using ELISA. *p < 0.05; **p < 0.01 (two-tailed Student’s t-test). Each gene expression level was measured by qPCR. The results represent single determination (day 0; n = 1) or the mean ± S.D. of triplicate determinations in separate wells of the culture plate (day 2, 7, and 14; n = 3) from a single experiment. The y-axis represents the relative expression level of each gene to that of the original ch-hepatocytes. Day 0 denotes the mRNA levels of isolated PXB-cells or thawed ch-hepatocytes. *p < 0.05; **p < 0.01 (two-tailed Student’s t-test).
(TIF)

S2 Fig Gene expression levels of human phase I and II enzymes in cFHHs and fresh adult h-hepatocytes (unplated). cFHHs were collected from chimeric mice transplanted with cells of donor A, B, and C. Fresh adult h-hepatocytes were collected from excess normal liver tissues of surgical liver resections from 4 donors. Each gene expression level was measured by qPCR. The results indicate the relative value to the average of cFHHs normalized by value of hGAPDH. The results of cFHHs indicate the mean ± S.D. of three different animals. The results of fresh adult h-hepatocytes indicate the mean ± S.D. of four donors from 25 to 61 years of age. The y-axis represents the relative expression level of each gene to the mean value of cFHHs.
(TIF)

S3 Fig MRP2 transporter activity in cFHHs. At day 16, cFHHs were incubated with HBSS containing 1.25 μM CDFDA for 5 min, and then incubated for up to 30 min in the presence or absence of Ca^2+^. CDF accumulation in bile canaliculi between cFHHs was observed as green spots. The bar denotes 100 μm.
(TIF)

S1 Table Primer sets. (DOCX)

S2 Table Altered expression of genes after 7 days at high density compared to 7 days at low density (day 7H > day 7L). (DOCX)
