# Supplementary material for: Culture density contributes to hepatic functions of fresh human hepatocytes isolated from chimeric mice with humanized livers: Novel, long-term, functional two-dimensional in vitro tool for developing new drugs
Source: PLoS One. 2020 Sep 11;15(9):e0237809. doi: 10.1371/journal.pone.0237809 (PMC7485858; doi:10.1371/journal.pone.0237809)
Supplement: S1 File — (DOCX) [file pone.0237809.s001.docx]

**Materials and Methods**

**Urea synthesis assay**

cFHHs (donor A) were cultured from day 1 in dHCGM. For the urea synthesis measurement at days 8, the medium was changed at days 6 and 7 in dHCGM containing GlutaMax™ (Thermo Fisher Scientific, Waltham, MA) instead of L-glutamine and removed FBS (GlutaMax medium). The culture medium at days 8 was collected and the concentration of urea was measured using QuantiChrom Urea Assay Kit (BioAssay systems, Hayward, CA). For the measurement at days 15, the medium was changed at days 13 and 14 in the GlutaMax medium. The culture medium at days 15 was collected and the concentration of urea was measured.

**Measurement of CYP activities using a cocktail mixture of probe substrates**

Sample preparation for the evaluation of the CYP1A2, CYP2C19, and CYP3A activities of cFHHs (Donor D) were conducted. The isolated (day 0) cFHHs were incubated in Williams’E medium containing Primary Hepatocyte Maintenance Supplements (Thermo Fisher Scientific, Waltham, MA) with a probe substrate at 37°C for 2 h, namely 50 µM phenacetin, 50 µM S-mephenytoin, and 5 µM midazolam for the assessment of the CYP1A2, CYP2C19, and CYP3A activities, respectively. The incubated solution was collected and the concentration of the metabolites (acetaminophen, 4’-hydroxy S-mephenytoin, and 1'-hydroxymidazolam) was measured by liquid chromatography-tandem mass spectrometry (LC-MS/MS). LC-MS/MS analysis was performed on HPLC LC20A system (Shimadzu Corporation Kyoto, Japan) and API 4000™ (AB Sciex Pte. Ltd., Framingham, MA) by Sumika Chemical Analysis Service, Ltd. (Osaka, Japan).

**Preparation of samples for mRNA expression analysis in fresh h-hepatocytes from human normal tissues**

Fresh h-hepatocytes isolated at ‎Hiroshima University Hospital were provided. Normal liver tissues were obtained from the resected liver of four patients (39- and 61-year-old men and 25- and 57-year-old women) after written receiving consent prior to surgery, in accordance with the 1975 Declaration of Helsinki. The hepatocytes were isolated via two-step collagenase perfusion and low-speed centrifugation [8]. They were then incubated in lysed in lysis buffer and stored in freezer until further use. Total RNA was isolated from each sample using RNeasy Micro Kit (Qiagen, Hilden, Germany). DNase treatment, cDNA synthesis, and qPCR analysis were conducted using the same procedure in the Materials and Methods. This study was approved by Utilization of Human Tissue Ethical Committee of PhoenixBio Co., Ltd. (0051).
