# Supplementary figures and images for: Culture density contributes to hepatic functions of fresh human hepatocytes isolated from chimeric mice with humanized livers: Novel, long-term, functional two-dimensional in vitro tool for developing new drugs
Source: PLoS One. 2020 Sep 11;15(9):e0237809. doi: 10.1371/journal.pone.0237809 (PMC7485858; doi:10.1371/journal.pone.0237809)

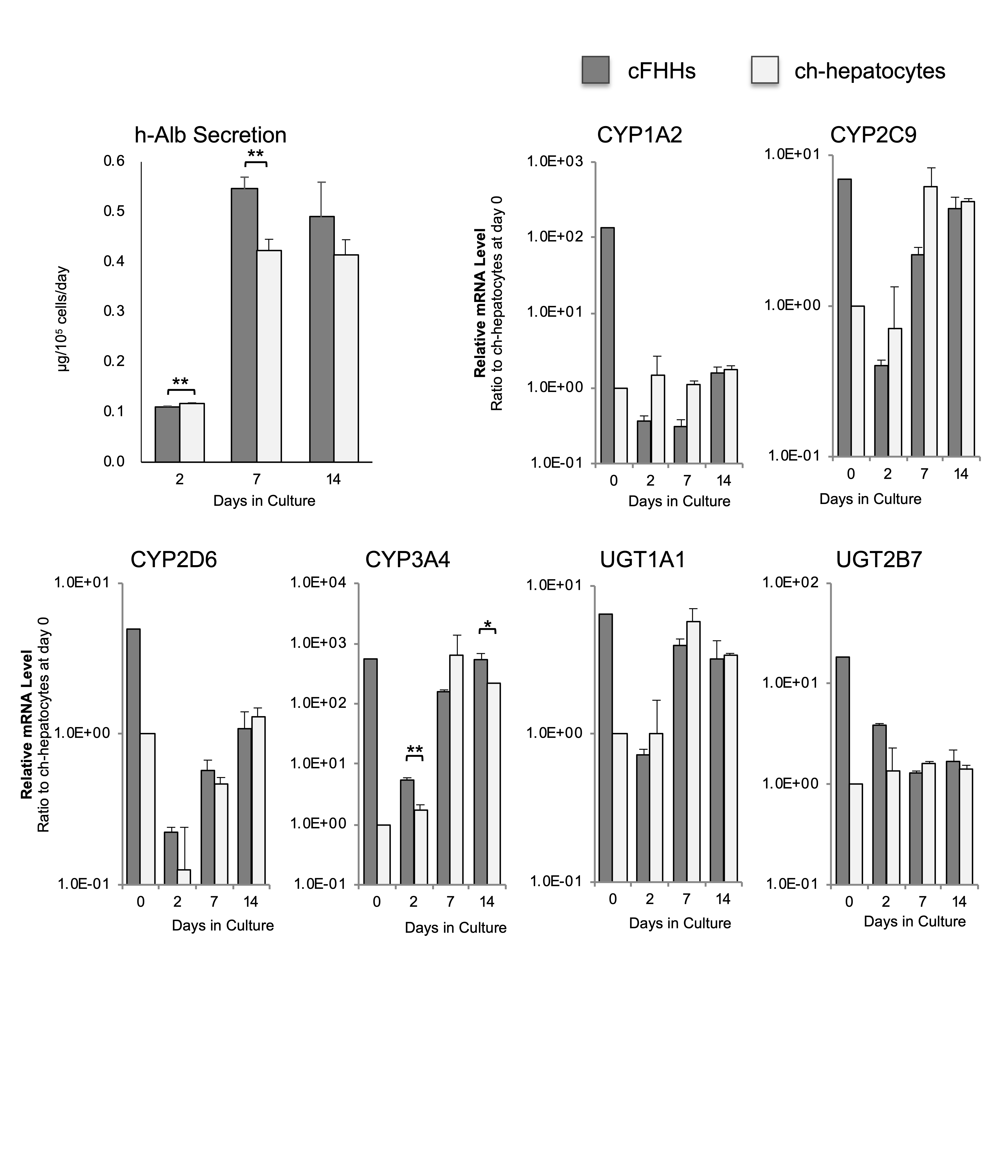

Supplement: S1 Fig — PXB-cells originating from donor A and original ch-hepatocytes (donor A) were plated at 2.13 × 10^5^ cells/cm2 and cultured for up to 14 days. h-Alb levels in the culture supernatant were measured using ELISA. *p < 0.05; **p < 0.01 (two-tailed Student’s t-test). Each gene expression level was measured by qPCR. The results represent single determination (day 0; n = 1) or the mean ± S.D. of triplicate determinations in separate wells of the culture plate (day 2, 7, and 14; n = 3) from a single experiment. The y-axis represents the relative expression level of each gene to that of the original ch-hepatocytes. Day 0 denotes the mRNA levels of isolated PXB-cells or thawed ch-hepatocytes. *p < 0.05; **p < 0.01 (two-tailed Student’s t-test). (TIF) [file pone.0237809.s002.tif]

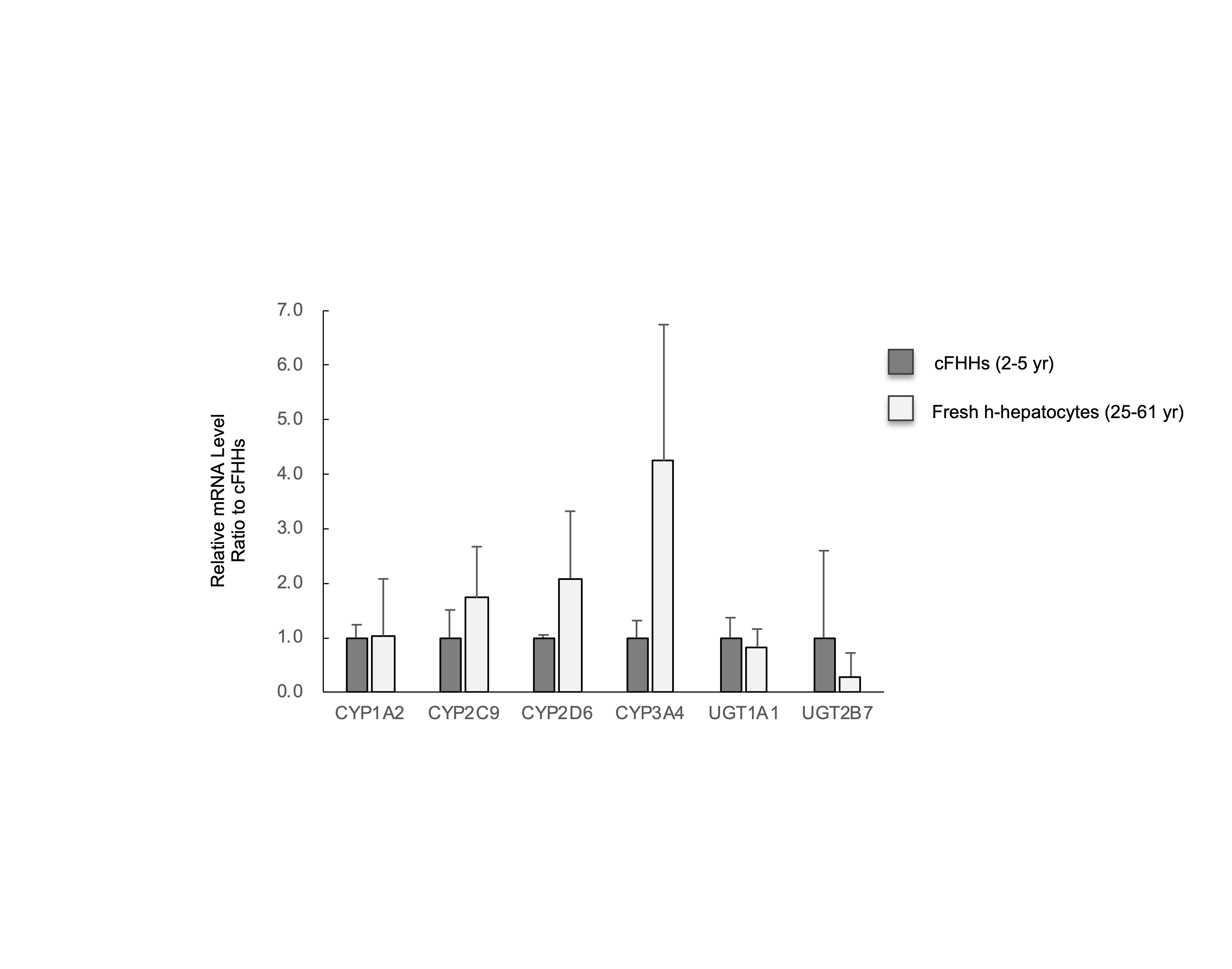

Supplement: S2 Fig — cFHHs were collected from chimeric mice transplanted with cells of donor A, B, and C. Fresh adult h-hepatocytes were collected from excess normal liver tissues of surgical liver resections from 4 donors. Each gene expression level was measured by qPCR. The results indicate the relative value to the average of cFHHs normalized by value of hGAPDH. The results of cFHHs indicate the mean ± S.D. of three different animals. The results of fresh adult h-hepatocytes indicate the mean ± S.D. of four donors from 25 to 61 years of age. The y-axis represents the relative expression level of each gene to the mean value of cFHHs. (TIF) [file pone.0237809.s003.tif]

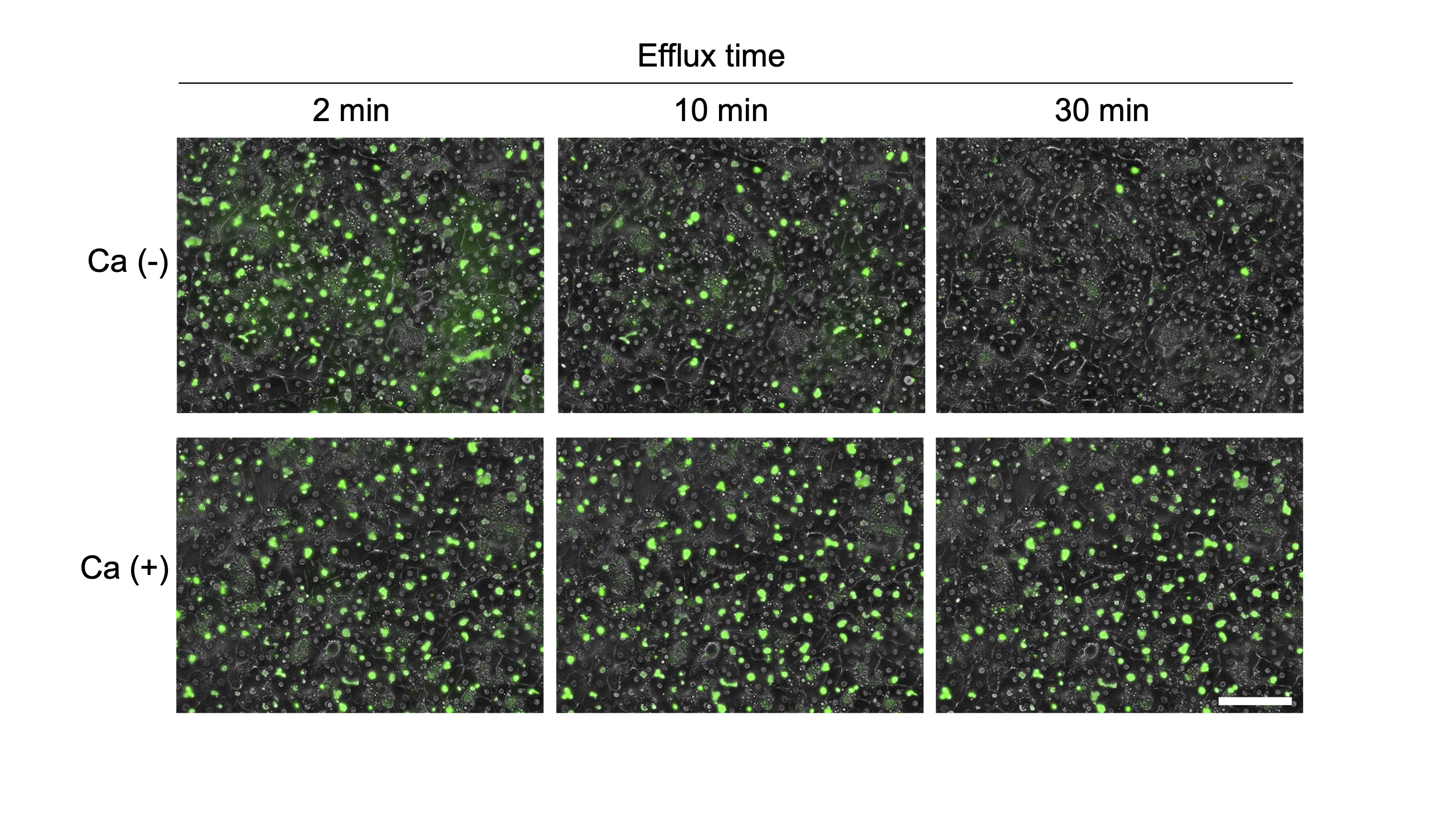

Supplement: S3 Fig — At day 16, cFHHs were incubated with HBSS containing 1.25 μM CDFDA for 5 min, and then incubated for up to 30 min in the presence or absence of Ca2+. CDF accumulation in bile canaliculi between cFHHs was observed as green spots. The bar denotes 100 μm. (TIF) [file pone.0237809.s004.tif]
