# Supplementary material for: Culture density contributes to hepatic functions of fresh human hepatocytes isolated from chimeric mice with humanized livers: Novel, long-term, functional two-dimensional in vitro tool for developing new drugs
Source: PLoS One. 2020 Sep 11;15(9):e0237809. doi: 10.1371/journal.pone.0237809 (PMC7485858; doi:10.1371/journal.pone.0237809)
Supplement: S1 Table — (DOCX) [file pone.0237809.s005.docx]

**S1 Table**. **Primer sets**

| **Gene name** | **F-primer** | **R-primer** |
| --- | --- | --- |
| hCYP1A1 | TCAACCATGACCAGAAGCTA | AAGATAATCACCTTCTCACTTAACAC |
| hCYP1A2 | GCTTCTACATCCCCAAGAAAT | ACCACTTGGCCAGGACT |
| hCYP2B6 | ACCCAACACACCAGCTTCCG | CAGGATTGAAGGCGTCTGGTTTTTC |
| hCYP2C9 | CCAGATCTGCAATAATTTTTCTC | CAAGCTTTCAATAGTAAATTCAGATG |
| hCYP2D6 | CTTGGACAAAGCCGTGA | GACAGCATTCAGCACCTC |
| hCYP2E1 | GGCTGAAGTAAAAGAGTATGTGTC | TTTCCTTCTCCATTTCCAC |
| hCYP3A4 | ACTGCCTTTTTTGGGAAATA | GGCTGTTGACCATCATAAAAG |
| hUGT1A1 | TTGATCCCAGTGGATGGC | ATGCTCCGTCTCTGATGTACAAC |
| hUGT2B7 | TGACATGAAGAAGTGGGATCAG | CAACATTTGGTAAGAGTGGATATGG |
| hOATP1B1 | TCATACTCTGTGAAAACAAATCAG | CAGACTGGTTCCCATTGAC |
| hOATP1B3 | CTCTGTTTGCTAAAATGTACGTG | GAAGAAATAATGGAAAATAGTCCAG |
| hBSEP | AAATATGCTTTTGGGTCATTG | GTCAGCTATGGCATCATTG |
| hMRP2 | TCCAACTGTGCTTCAAGC | GGCATCCACAGACATCAG |
| hHNF4α | GACCGCCAGTATGACTCG | CGTTGGTTCCCATATGTTCC |
| hCAR | ATTGAAGATGGAGCCCG | GAGCTGCAGTTTTCGTAGTG |
| hPXR | AGCTACTCCTTGATCGATCC | AAAGTCAGCATGGTTCCAG |
| hFXR | ATGGATTCATATAACAAACAGAGG | GCTTTTTTGTGAATTCTACAAGAAC |
| hGAPDH | GGAGTCAACGGATTTGGT | AAGATGGTGATGGGATTTCCA |
| mCyp1a2 | CTCCTTTAAGGAAAACCCAACCACC | ACAACACTGGGTCAGAATCTCATTG |
| mCyp3a11 | CTAAGCAGAAGCACCGAGTGG | TTTCTGGATATCAGGGTGAGTGG |
