# Supplementary material for: Culture density contributes to hepatic functions of fresh human hepatocytes isolated from chimeric mice with humanized livers: Novel, long-term, functional two-dimensional in vitro tool for developing new drugs
Source: PLoS One. 2020 Sep 11;15(9):e0237809. doi: 10.1371/journal.pone.0237809 (PMC7485858; doi:10.1371/journal.pone.0237809)
Supplement: S2 Table — (DOCX) [file pone.0237809.s006.docx]

**S2 Table.** **Altered expression of** **genes after 7 days at high density compared to 7 days at low density**.

| Fold change | Transcription factors | |  | Phase I enzymes | |  | Phase II enzymes | | | |  | Transporters | |
| --- | --- | --- | --- | --- | --- | --- | --- | --- | --- | --- | --- | --- | --- |
|  | NR | Others |  | CYP | FMO |  | UGT | SULT | GST | NAT |  | ABC | SLC |
| 2-4 | 1H3 (LXRa),  1I2 (PXR),  3C2 (AR),  5A2 (LRH-1) | HNF4A |  | 2C18, 2J2,  3A4, 3A43 | 3 |  | 1A6,3A1 | 1A1 | A4 |  |  | A8, C2 (MRP2),  C3 (MRP3),  C6 (MRP6)/C6P1/C6P2, G2 | 2A2 (GLUT2), 7A2 (CAT2),  15A1 (PEPT1), 16A10 (MCT10), 19A3 (THTR2), 22A18, 22A25, 23A1, 25A13 (AGC2),  25A15 (ORC1), 25A20 (CAC), 25A3, 25A33, 25A42, 30A10, 31A1, 35D1, 44A1 (CTL1),  46A3, 50A1 47A1 (MATE1) |
| 4-6 | 0B2 (SHP1) |  |  | 2W1, 4F3, 4F12,  8B1, 39A1 |  |  | 2A3 | 2A1 |  | 8 |  | C6P1, G5 | 13A5 (NACT), 17A3 (NPT4),  22A3 (OCT3), 38A4 (ATA3) |
| >6 | 1I3 (CAR) |  |  | 2A6, 2A7, 2B6,  2C8, 2C9, 2C19,  2D6/2D7, 2E1, 3A7, 4A11, 4F2, 7A1, 26A1 | 1,  5 |  | 1A8/1A9, 2B4, 2B15, 2B28 |  |  |  |  | A6, B4 (MDR3),  G8 (GBD4) | 6A1 (GAT-1), 10A1 (NTCP), 17A4, 1A2 (GLT1),  22A1 (OCT1), 22A7 (OAT2), 25A47, 28A1 (CNT1), 38A3, 39A5, 51A (OST alpha),  O1B1 (OATP1B1),  O1B3 (OATP1B3) |
